# Chronic Hyperglycaemia Inhibits Tricarboxylic Acid Cycle in Rat Cardiomyoblasts Overexpressing Glucose Transporter Type 4

**DOI:** 10.3390/ijms23137255

**Published:** 2022-06-29

**Authors:** Bernd Stratmann, Britta Eggers, Yvonne Mattern, Tayana Silva de Carvalho, Katrin Marcus, Diethelm Tschoepe

**Affiliations:** 1 Herz- and Diabeteszentrum NRW, Diabeteszentrum, Ruhr Universität Bochum, 32545 Bad Oeynhausen, Germany; ymattern@hdz-nrw.de (Y.M.); tsdcgrosserichter@hdz-nrw.de (T.S.d.C.); diethelm.tschoepe@ruhr-uni-bochum.de (D.T.); 2 Medizinisches Proteom-Center, Centre for Translational and Behavioural Neurosciences, Medical Faculty, Ruhr-University Bochum, 44801 Bochum, Germany; britta.eggers@rub.de (B.E.); katrin.marcus@rub.de (K.M.); 3 Medical Proteome Analysis, Centre for Protein Diagnostics (PRODI), Ruhr-University Bochum, 44801 Bochum, Germany; 4 Stiftung DHD (Der herzkranke Diabetiker) Stiftung in der Deutschen Diabetes-Stiftung, 32545 Bad Oeynhausen, Germany

**Keywords:** diabetic cardiomyopathy, TCA cycle, fumarate, apoptosis, metabolic starvation

## Abstract

An oversupply of nutrients with a loss of metabolic flexibility and subsequent cardiac dysfunction are hallmarks of diabetic cardiomyopathy. Even if excess substrate is offered, the heart suffers energy depletion as metabolic fluxes are diminished. To study the effects of a high glucose supply, a stably glucose transporter type 4 (GLUT4)-overexpressing cell line presenting an onset of diabetic cardiomyopathy-like phenotype was established. Long-term hyperglycaemia effects were analysed. Rat cardiomyoblasts overexpressing GLUT4 (H9C2KE2) were cultured under normo- and hyperglycaemic conditions for long-term. Expression profiles of several proteins were compared to non-transfected H9C2 cells (H9C2) using RT-qPCR, proteomics-based analysis, or Western blotting. GLUT4 surface analysis, glucose uptake, and cell morphology changes as well as apoptosis/necrosis measurements were performed using flow cytometry. Additionally, brain natriuretic peptide (BNP) levels, reactive oxygen species (ROS) formation, glucose consumption, and lactate production were quantified. Long-term hyperglycaemia in H9C2KE2 cells induced increased GLUT4 presence on the cell surface and was associated with exaggerated glucose influx and lactate production. On the metabolic level, hyperglycaemia affected the tricarboxylic acid (TCA) cycle with accumulation of fumarate. This was associated with increased BNP-levels, oxidative stress, and lower antioxidant response, resulting in pronounced apoptosis and necrosis. Chronic glucose overload in cardiomyoblasts induced by GLUT4 overexpression and hyperglycaemia resulted in metabolically stimulated proteome profile changes and metabolic alterations on the TCA level.

## 1. Introduction

Diabetic cardiomyopathy (DC) is characterised by marked structural and morphological changes at the myocardial level, resulting in left ventricular dysfunction [1]. It develops independently from further cardiac risk factors such as hypertension or coronary artery disease that progresses to heart failure (HF) as a secondary manifestation of diabetes [1]. In general, this patient group has a high mortality, corresponding to 1% of the cases among all patients diagnosed with diabetes [2,3]. In this context, diabetes mellitus per se is a cardiovascular risk factor and multiplies the risk for heart failure [3]. Although the mechanisms leading to DC are not fully understood, it is well-accepted that the combination of hyperglycaemia, reactive glucose metabolites such as methylglyoxal, and increased formation of advanced glycation end products (AGEs) in cardiomyocytes and endothelial cells contribute to the development of the disease [2,4]. AGEs stimulate structural, functional, and transcriptional changes. As a consequence of glucose and glycation overflow, the detoxifying enzymes progressively fail, limiting the glyoxalase function in the methylglyoxal elimination process. Insufficient activation of endogenous repair mechanisms results in mitochondrial dysfunction, increased glycation, and oxidative stress [5]. The combination of hyperglycaemic state and increased methylglyoxal concentration prolongs the glucose transporter presence at the cell surface and increases glucose influx, which is insulin-independent [6,7]. This state triggers a vicious cycle of glucose intoxication with subsequent glycation [6,7], resulting in oxidative and mitochondrial stress [8]. Glucose overflow induces impaired transcription of metabolic enzymes and structural molecules [6,7] and alters the mitochondrial function by post-translational modification [8]. These findings suggest an important role of glucose overflow in metabolic-induced organ damage.

In addition, and predominantly in later stages of metabolic impairment, insulin resistance supports a metabolic shift increasing the β-oxidation, intracellular fatty acid concentration, and reactive oxygen species production [2]. These events contribute to cardiomyocyte malfunction and death, cardiac hypertrophy, and fibrosis [2]. At this level, further alteration at Ca^2+^ signalling and titin phosphorylation are also observed, providing a beneficial scenario for the development of HF [2].

Another important metabolic pathway possibly associated with DC development is the tricarboxylic acid (TCA) cycle [9]. This cycle is responsible for producing reduced nicotinamide adenine dinucleotide (NADH) through the oxidation of acetyl-coenzyme A (acetyl-CoA) derived from carbohydrates, fatty acids, and proteins [10]. In type 1 diabetes mellitus, this cycle is highly altered, contributing to the development of cardiovascular autonomic neuropathy [11]. A similar effect was observed in type 2 diabetes mellitus, resulting in an inefficient mitochondrial TCA cycle influx and reduced energy yield in skeletal muscle [12]. Following this dysfunction, high levels of insulin lead to lower expression of citrate synthase, one of the first enzymes responsible for initiating the TCA cycle activity [12]. This reduction in the diabetic mitochondria influx is based on profound posttranscriptional modifications of all TCA cycle enzymes [13].

Little is known about the TCA cycle response in DC. Particularly, full changes at the proteome levels or the gene and protein expression of these enzymes in diabetic myocytes have not been investigated so far. One recent paper proved that myocardial glucose utilisation is critical in diabetic cardiomyopathy. Restoring glucose uptake was associated with impairments of mitochondrial energy gain, exerting new effects of glucose in diabetic heart metabolism [8]. To further address this, the present study aimed to develop a suitable model for reproducing the onset of DC. A model that could mimic the hyperglycaemic state, the overexpression of glucose transporter type 4 (GLUT4), and, by glucose overload, the inactivation of the glyoxalase system in cardiomyocytes.

Therefore, we established a rat cardiomyoblast cell line which stably overexpressed GLUT4 (H9C2KE2). These cells were exposed to hyperglycaemia for nine months to develop a DC-onset-like phenotype. Additionally, metabolic alterations were monitored concerning the TCA cycle under increased glucose influx in cardiomyoblasts.

## 2. Results

### 2.1. Overexpression of GLUT4 in Hyperglycaemic H9C2 as a Possible Model for Mimicking the Onset of DC

**Figure 1:**
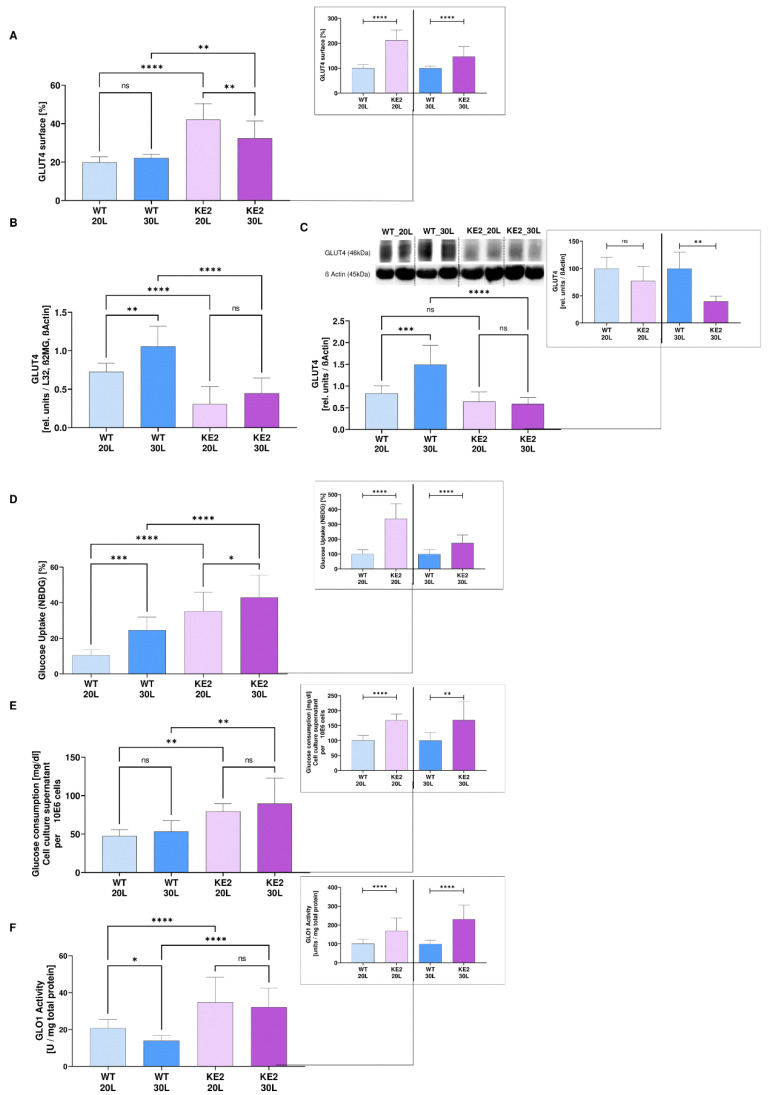
Characterisation of the DC model, H9C2KE2 cells exposed to 20 mM or 30 mM glucose. (**A**): Percentage of GLUT4 overexpression at the surface of H9C2KE2 cells (N = 6); (**B**): *GLUT4* gene expression (N = 6); (**C**): GLUT4 protein expression (N = 6); (**D**): Percentage of Glucose uptake (NBDG) (N = 11); (**E**) Glucose consumption in cell culture supernatant (N = 3); (**F**) Glo1 activity (N = 8). Representative Western blots are also shown. Data are shown as mean ± SD values described as WT20L vs. KE220L and WT30L vs. KE230L with * *p* < 0.05, ** *p* < 0.01, *** *p* < 0.001, or **** *p* < 0.0001 for showing the significance. Shapiro-Wilk test was applied for testing the data normality distribution. For *t*-test analysis, an unpaired test for all normally distributed data and Mann-Whitney for all abnormally distributed data was used. (WTL–Wild-type cells exposed for long-term/KE2L–GLUT4 overexpressing cells exposed for long-term).

To measure whether the overexpression of GLUT4 in H9C2 can be a suitable model for mimicking the onset of DC, cells were chronically exposed to 20 mM or 30 mM glucose for nine months (long-term exposition, L). These glucose concentrations resemble normo- and hyperglycaemia in this cell type [14]. After this time, the GLUT4 presence on the cell surface significantly increased in normo- and hyperglycaemic H9C2KE2 cells if compared to wild type cells (2-fold more, WT30L vs. KE230L, *p* < 0.0001/1.4-fold more, WT20L vs. KE220L, *p* < 0.0001, *t*-test) (Figure 1A). However, in terms of gene expression and protein expression after long-term hyperglycaemia, a significant reduction of GLUT4 expression was detected (gene: 2-fold less, WT30L vs. KE230L, *p* < 0.0001, *t*-test; protein 3-fold less, WT30L vs. KE230L, *p* < 0.01, *t*-test) (Figure 1B,C, respectively).

By measuring the 2-NBDG-uptake via flow cytometry, H9C2KE2 cells proved to more effectively increase the glucose uptake regardless of the amount (3-fold more; WT30L vs. KE230L/1.7-fold more, *p* < 0.0001, WT20L vs. KE220L, *p* < 0.0001, *t*-test) (Figure 1D). Concomitantly, the glucose consumption similarly increased for the same cells (1.6-fold more, WT30L vs. KE230L, *p* < 0.0001/1.6-fold more, WT20L vs. KE220L, *p* < 0.01, *t*-test) (Figure 1E). In terms of L-lactate production, H9C2KE2 cells doubled the production after the long-term (2-fold more, WT30L vs. KE230L, *p* < 0.0001/2-fold more, WT20L vs. KE220L, *p* < 0.0001, one-way ANOVA) (Figure S1A).

The overexpression of GLUT4 markedly influenced the enzymatic activity of glyoxalase 1 (GLO1) after chronic exposure to glucose (regardless of the concentration) (Figure 1F) (1.6-fold more, WT20L vs. KE220L, *p* < 0.0001/2.3-fold more, WT30L vs. KE230L, *p* < 0.0001, *t*-test). However, a similar increment was not detected in the protein expression and gene expression. The *GLO1* gene expression showed a gradual reduction when compared to the WT cells (1.5-fold less, WT20L vs. KE220L, *p* < 0.0001/1.3-fold less, WT30L vs. KE230L, *p* < 0.05, one-way ANOVA), followed by no alterations at the protein expression levels (Figure S1B,C, respectively). Moderate changes were found in terms of D-lactate production (Figure S1D). However, this alteration was just effective when normoglycaemic H9C2KE2 cells were compared with wild-type cells (3-fold, *p* < 0.001, one-way ANOVA).

In terms of morphology and protein content, normo- and hyperglycaemic H9C2KE2 cells presented with significantly increased size (1.4-fold, *p* < 0.0001, one-way ANOVA), with greater granularity (1.5-fold, *p* < 0.0001, one-way ANOVA) and higher total protein content (1.4-fold, *p* < 0.0001, and *p* < 0.001, one-way ANOVA, respectively) when compared with WT cells (Figure S1E,F,G, respectively). 

### 2.2. Glucose Overflow Might Inhibit the Last Steps of the TCA Cycle

A proteomic-based analysis was performed, quantifying a total of 2654 proteins. Among these quantified proteins, 21 (from 22) TCA cycle annotated proteins could be detected. To estimate their absolute abundance in the different sample groups, we used iBAQ, the intensity-based absolute quantification option provided in MaxQuant (Table S2).

**Figure 2:**
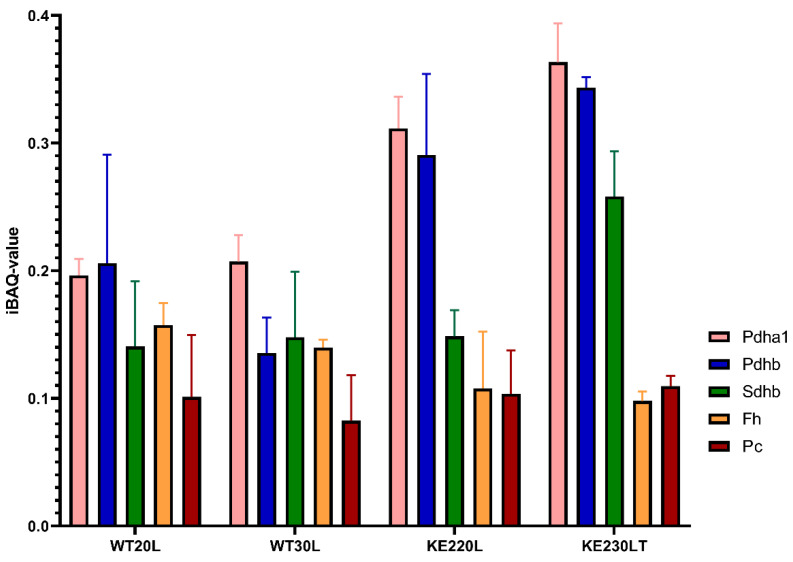
Proteomic changes in H9C2KE2 cells at the TCA cycle level including PDH-reaction. IBAQ values represent the relative abundance of detected proteins within the respective analysed set. Pdha1 = pyruvate dehydrogenase complex A, Pdhb = Pyruvate dehydrogenase complex B, Sdhb = succinate dehydrogenase complex B, Fh = fumarase, Pc = pyruvate carboxylase, N = 3, data are shown as mean ± SD values (WTL–Wild-type cells exposed for long-term/KE2L–GLUT4 overexpressing cells exposed for long-term).

Key regulatory enzymes were analysed further (Figure 2). The abundance of pyruvate carboxylase was slightly higher in hyperglycaemic H9C2KE2 cells when compared with hyperglycaemic WT cells. However, in all H9C2KE2 cells, a high abundance of pyruvate carboxylase could be observed regardless of the level of glucose, compared to WT cells. The abundance of fumarase was consistently lower in H9C2KE2 cells, regardless of the level of glucose, when compared with WT cells. However, succinate dehydrogenase presented with lower abundance in hyperglycaemic H9C2KE2 cells when compared with hyperglycaemic WT cells.

**Figure 3:**
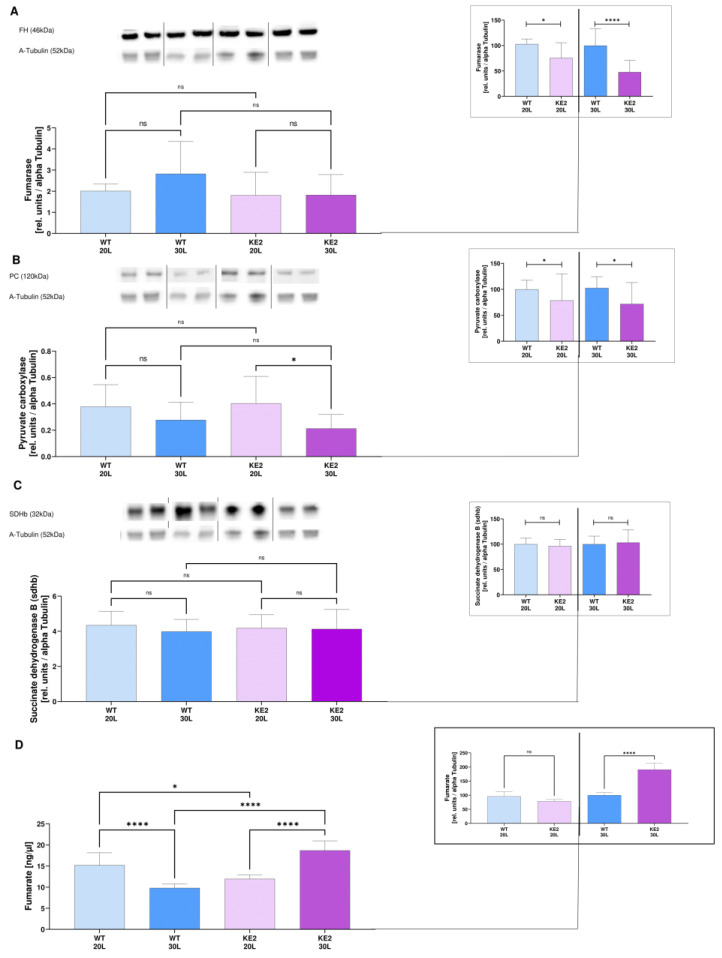
Expression of TCA cycle enzymes in H9C2KE2 cells. (**A**): Fumarase protein expression (N = 6); (**B**): Pyruvate carboxylase protein expression (N = 8); (**C**): Succinate Dehydrogenase B protein expression (N = 8); (**D**): Fumarate levels (N = 6). Representative Western blots are also shown. Data are shown as mean ± SD values described as KE220L vs. KE230L and WT30L vs. KE230L with * *p* < 0.05, **** *p* < 0.0001 for showing the significance. A Shapiro-Wilk test was applied for testing the data normality distribution. For *t*-test analysis, an unpaired test for all normally distributed data and a Mann-Whitney for all abnormally distributed data was used. (WTL–Wild-type cells exposed for long-term/KE2L–GLUT4 overexpressing cells exposed for long-term).

By Western Blotting, we found that H9C2KE2, regardless of the glucose concentration, had less protein expression of fumarase when compared with WT cells (1.36-fold less, WT20L vs. KE220L, *p* < 0.05/2.10-fold less, WT30L vs. KE230L, *p* < 0.001, *t*-test) (Figure 3A).

The protein expression of pyruvate carboxylase followed the same profile as observed in fumarase, when compared with WT cells (1.27-fold less, WT20L vs. KE220L, *p* < 0.05/1.39-fold less, WT30L vs. KE230L, *p* < 0.05, *t*-test) (Figure 3B). Succinate dehydrogenase B was unaffected in chronically hyperglycaemic H9C2KE2 cells when compared with normoglycaemic cells (KE220L vs. KE230L, *p* = ns, one-way ANOVA) or WT30L cells (*p* = ns, *t*-test) (Figure 3C).

In terms of enzymatic activity, we found that fumarate concentrations were significantly increased, particularly after chronical hyperglycaemic in H9C2KE2 cells, when compared with WT cells (1.22-fold less, WT20L vs. KE220L, *p* < 0.01/1.9-fold more, WT30L vs. KE230L, *p* < 0.001, *t*-test) (Figure 3D). 

### 2.3. GLUT4 Overexpression Followed by Hyperglycaemia—Effects on Glycolysis

**Figure 4:**
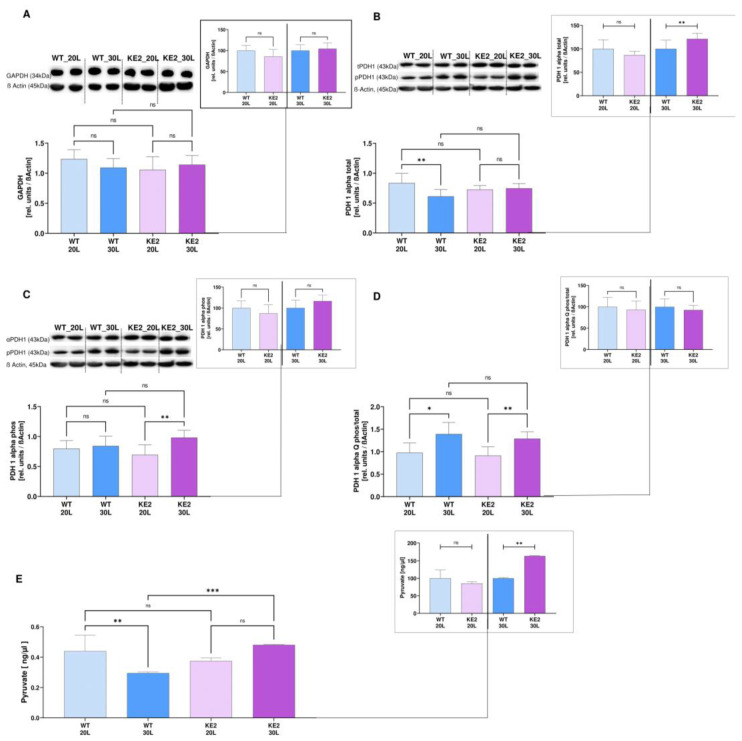
Protein expression and enzymatic activity of proteins associated with glycolysis in H9C2KE2 cells. (**A**): Protein expression of GAPDH (N = 4); (**B**): Protein expression of total PDH1α protein (N = 6); (**C**): Protein expression of phosphorylated PDH1α protein (N = 6); (**D**): Protein expression ratio of phosphorylated/total PDH1α protein; (**E**): Pyruvate levels (N = 6). Representative Western blots are also shown. Data are shown as mean ± SD values described as KE220L vs. KE230L, WT20L vs. KE220L, and WT30L vs. KE230L with * *p* < 0.05, ** *p* < 0.01, or *** *p* < 0.001 for showing the significance. Shapiro-Wilk test was applied for testing the data normality distribution. For *t*-test analysis, an unpaired test for all normally distributed data and a Mann-Whitney for all abnormally distributed data was used. (WTL–Wild-type cells exposed for long-term/KE2L–GLUT4 overexpressing cells exposed for long-term).

Following the enzymatic evaluation, the next step was to closely control proteins associated with these enzymes, particularly proteins related to glycolysis. We found that GAPDH was upregulated at the gene expression level in hyperglycaemic H9C2KE2 cells, particularly when compared among the glucose concentrations (1.4-fold more, WT30L vs. KE230L, *p* < 0.05/1.4-fold more, KE220L vs. KE230L, *p* < 0.05, one-way ANOVA) (Figure S2A). However, this regulation did not extrapolate into protein expression level (Figure 4A).

Interestingly, the gene expression of *PDH1α* in H9C2KE2 cells statistically increased in all the H9C2KE2 cells exposed to glucose when compared to WT cells (1.38-fold more, WT20L vs. KE220L, *p* < 0.01/1.4-fold more, WT30L vs. KE230L, *p* < 0.01, one-way ANOVA) (Figure S2B). The total PDH1α protein expression did not follow gene expression at 20 mM glucose (WT20L vs. KE220L, *p* > 0.05); however, at hyperglycaemia it was significantly increased (1.2-fold more, WT30L vs. KE230L, *p* < 0.001, *t*-test) (Figure 4B). The grade of phosphorylation was significantly different between culture conditions. In hyperglycaemia, increased phosphorylation occurred: (WT20L vs. WT30L, *p* < 0.05; KE220L vs. KE230L, *p* < 0.01, *t*-test) but phosphorylation grade was independent of cell type (Figure 4C,D).

In terms of pyruvate, this increased production persisted only when comparing hyperglycaemic H9C2KE2 cells with hyperglycaemic WT cells (1.6-fold more, WT30L vs. KE230L, *p* < 0.01, *t*-test) after the long-term exposition (Figure 4E).

### 2.4. The Accumulation of Fumarate in H9C2KE2 Cells Induces Oxidative Stress

**Figure 5:**
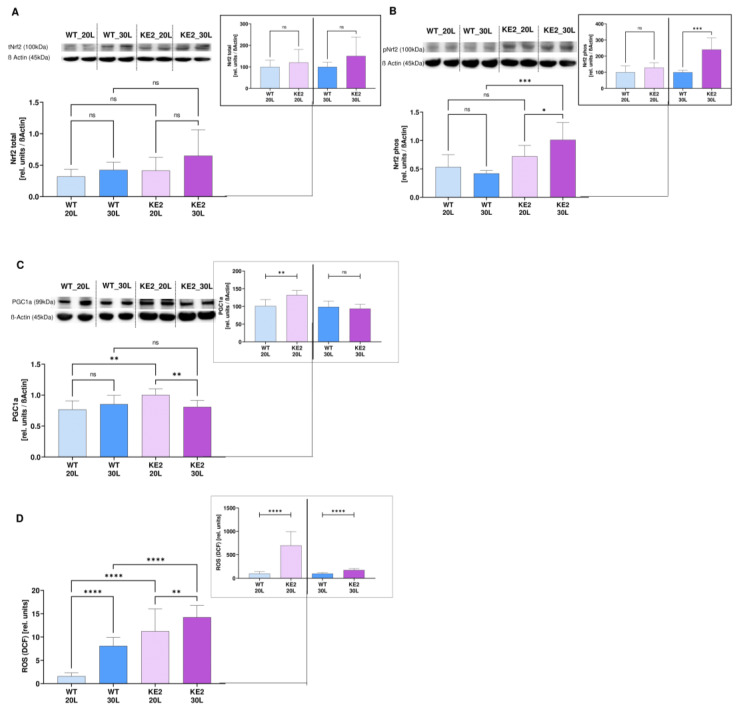
Consequences of fumarate accumulation in hyperglycaemic H9C2KE2 cells. (**A**): Total Nrf2 protein expression (N = 6); (**B**): phosphorylated Nrf2 protein expression (N = 8); (**C**): Protein expression of PGC1α (N = 5); (**D**): ROS production (N = 17). Representative Western blots are also shown. Data are shown as mean ± SD values de-scribed as KE220L vs. KE230L, WT20L vs. KE202L, and WT30L vs. KE230L with * *p* < 0.05, ** *p* < 0.01, *** *p* < 0.001, or **** *p* < 0.0001 for showing the significance. Shapiro-Wilk test was applied for testing the data normality distribution. For *t*-test analysis, an unpaired test for all normally distributed data and a Mann-Whitney for all abnormally distributed data was used. (WTL–Wild-type cells exposed for long-term/KE2L–GLUT4 overexpressing cells exposed for long-term).

Fumarate accumulation in H9C2KE2 cells resulted in an upregulation of *NRF2* gene expression when compared with WT cells (1.3-fold more, WT20 vs. KE220, *p* < 0.01/1.5-fold more, WT30L vs. KE230L, *p* < 0.0001, one-way ANOVA), and was even more pronounced when compared between the hyper- and normoglycaemic H9C2KE2 cells (2.15-fold more, *p* < 0.0001, one-way ANOVA) (Figure S3A). However, only the phosphorylation of Nrf2 was upregulated by long-term hyperglycaemia when compared with WT cells (2-fold more, WT30L vs. KE230L, *p* < 0.001, *t*-test) or among to the H9C2KE2 cells (*p* < 0.05) (Figure 5A,B). These changes were not extended to *KEAP1* (WT20L vs. KE220L, *p* > 0.05/1.4-fold more, WT30L vs. KE230L, *p* < 0.05, one-way ANOVA), showing that the gene expression of this protein was not able to completely inhibit the Nrf2 activity (Figure S3B).

The protein expression of PGC1α was lower in hyperglycaemic cells when compared with normoglycaemic H9C2KE2 cells (1.25-fold less, KE220L vs. KE230L, *p* < 0.01, one-way ANOVA) but was higher when compared with WT cells. This might occur because normoglycaemic H9C2KE2 cells induce a significant overexpression of this protein (*p* < 0.01, when compared with normoglycaemic WT cells). It is expected that H9C2KE2 cells lose antioxidant protection upon stimulation with high levels of glucose (Figure 5C).

The reactive oxygen species production was massively and significantly increased in H9C2KE2 cells when compared with WT cells (6.9-fold more, WT20L vs. KE220, *p* < 0.0001; 1.7-fold more, WT30L vs. KE230L, *p* < 0.0001, *t*-test) or among the H9C2KE2 (*p* < 0.01) cells conditions after long-term hyperglycaemia (Figure 5D). Additionally, we observed an increased gene expression of NAD(p)H dehydrogenase (quinone)-1 (*NQO1*) in normoglycaemic and hyperglycaemic H9C2KE2 cells when compared with WT cells in the same conditions (3.5-fold more, WT20L vs. KE220L, *p* < 0.01/4-fold more, WT30L vs. KE230L, *p* < 0.01, one-way ANOVA) (Figure S3C). However, for the gene expression of Haeme Oxygenase 1 (*HO1*), only hyperglycaemic H9C2KE2 cells showed an upregulation of this gene when compared with hyperglycaemic WT cells (2-fold more, WT30L vs. KE230L, *p* < 0.01, one-way ANOVA) (Figure S3D). Therefore, these results indicated that in hyperglycaemic H9C2KE2 cells, further pro-oxidative pathways are activated.

### 2.5. The Accumulation of Fumarate in H9C2KE2 Cells Activates Cell Death

**Figure 6:**
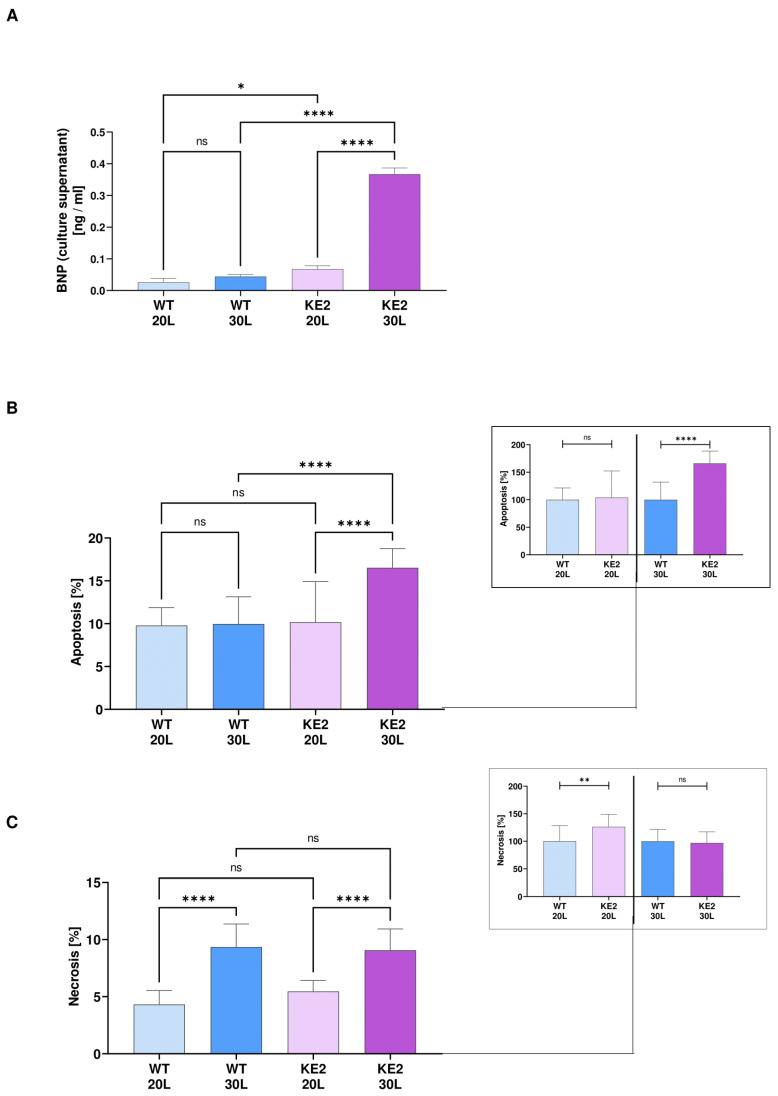
Metabolic and pro-inflammatory response in H9C2KE2 cells. (**A**): BNP levels in the cell culture supernatant (N = 3); (**B**): Apoptosis quantification (N = 6); (**C**): Necrosis quantification (N = 6). Data are shown as mean ± SD values described as KE220L vs. KE230L, WT20L vs. KE220L, and WT30L vs. KE230L with * *p* < 0.05, ** *p* < 0.01, or **** *p* < 0.0001 for showing the significance (N = 3). Shapiro-Wilk test was applied for testing the data normality distribution. For *t*-test analysis, an unpaired test for all normally distributed data and a Mann-Whitney for all abnormally distributed data was used. (WTL–Wild-type cells exposed for long-term/KE2L–GLUT4 overexpressing cells exposed for long-term).

Hyperglycaemic H9C2KE2 cells showed significant overexpression of BNP when compared with hyperglycaemic WT cells (8.3-fold more, WT30L vs. KE230L, *p* < 0.0001) or among the H9C2KE2 cells (2.5-fold more, *p* < 0.0001, one-way ANOVA) (Figure 6A).

The profound changes induced by overexpression of GLUT4 associated with hyperglycaemia could no longer avoid the activation of cell death mechanisms, proved by increased levels of apoptosis (1.7-fold more, WT30L vs. KE230L, *p* < 0.0001/1.6-fold more, KE220L vs. KE230L, *p* < 0.0001, one-way ANOVA) (Figure 6B). However, in the case of necrosis, this alteration is more likely related to hyperglycaemia than to GLUT4 overexpression itself, as hyperglycaemic H9C2KE2 and WT cells showed a significant increase of necrosis events when compared with the same cells under normoglycaemic levels (2-fold more, WT20L vs. WT30L, *p* < 0.0001; 2.25-fold more, KE220L vs. KE230L, *p* < 0.0001, one-way ANOVA) (Figure 6C). However, only normoglycaemic KE2 cells had higher events when compared with normoglycaemic WT cells (1.2-fold more, WT20L vs. KE220L, *p* < 0.01; *t*-test). 

Therefore, considering all the structural, metabolic, and molecular changes induced by overexpression of GLUT4 in H9C2KE2 cells exposed to glucose, we have a basis for indicating that H9C2KE2 can be a suitable model for mimicking the metabolically induced cardiomyopathy.

## 3. Discussion

In H9C2 cardiomyoblasts, chronic hyperglycaemia leads to increased glucose influx. In our model of GLUT4 overexpressing H9C2 cells, this is even more pronounced, although GLUT4 expression per se is downregulated on mRNA as well as on the protein level. Prolonged presentation of GLUT4 on the cell surface by reduced internalisation was detected and might explain increased glucose influx and consumption as shown in our model. Reactive glucose metabolites originating from glycolysis overflow induce increased detoxifying mechanisms, as shown by increased glyoxalase activity. These findings suggest a direct impact of glucose influx on cardiomyocyte fate, as it is associated with generation of oxidative stress and subsequent cell death. This effect was insulin-independent, suggesting deleterious cardiac effects beyond the classical model of insulin resistance as being an exclusive driver of DC. Therefore, we assume that our model may mimic the early phase in which surplus of nutrients and subsequent glycation boost the onset of the disease.

Previously we have demonstrated a deleterious effect of glucose overflow in L6 myoblasts and identified the reactive dicarbonyl methylglyoxal as being involved in this pathology. Methylglyoxal stress prolonged GLUT4 presence at the cell surface of myoblasts [6]. This causes exaggerated glucose influx and high systemic glucose availability, resulting in activation of cell death mechanisms [6]. To analyse whether this myoblast-derived hypothesis is valuable in cardiomyoblasts, the well-established H9C2 cell culture model was adapted for this purpose [14]. GLUT4-overexpressing H9C2 cells (H9C2KE2) were developed to study the increased glucose uptake. By inducing this high-glucose influx, we assume increased intracellular methylglyoxal (MG) production from overflow in glycolysis, as shown before in L6 myoblasts [6]. H9C2KE2 cells react upon methylglyoxal stress via direct incubation with methylglyoxal with increased glucose uptake in a similar amount as if treated with insulin. This previous observation adds a new viewpoint to early pathologic mechanisms resulting in diabetes-induced cardiomyocyte damage and the role of reactive glucose metabolites in this scenario [15]. Increased glucose uptake induced by hyperglycaemia leads to impairments in glycolysis, generating reactive glucose metabolites such as methylglyoxal which then prolong GLUT4 presence on the cell surface, allowing for even more glucose to enter the cell. This is a self-accelerating vicious cycle leading to glucotoxicity influencing in a second instance metabolic pathways for energy generation, as proven by our data. Therefore, the classical view of cardiomyopathy as being induced by insulin resistance and low uptake of metabolites may be questioned in hyperglycaemia; besides the amount of GLUT4, its functionality and surface presentation should be recognised as being involved in the pathogenesis of a metabolically induced cardiomyocyte dysfunction, at least in early stages.

The morphological changes observed in H9C2KE2 cells also indicated that this model could mimic the structural changes associated with the onset of DC. These cells showed increased size and granularity as a result of enhanced protein synthesis and turnover. Long-term effects of high glucose and fatty acids on cardiomyocytes result in hypertrophy, particularly via upregulation of atrial natriuretic peptide (ANP), connective tissue growth factor, and α-skeletal actin gene expression [16]. BNP levels are a primary marker of cardiac dysfunction [17,18,19], especially because BNP activates lipid peroxidation and apoptosis, which are important inducers of heart failure at the level of the myocardium. Interestingly, the increase in the levels of BNP in DC is not fully understood, considering that most of these patients develop heart failure from the diabetic condition instead of having previous cardiac dysfunction [20]. Hence, we were the first to study an increased level of BNP under such conditions, proving that hyperglycaemic H9C2KE2 cells have a disturbed myocardial performance.

Glucose overload by GLUT4 overexpression can worsen the mitochondrial respiratory chain capacity of myocardial cells, yielding less efficient energy production. This effect was mainly driven by glycosylation of the transcription factor specificity protein 1 (SP1), a is master regulator of mitochondrial protein expression, identifying mitochondria as a major target of glucotoxicity [8]. To answer the question of whether increased glucose influx affects key metabolic pathways, we designed our GLUT4-overexpressing H9C2 cells based on previous experiences with GLUT4-overexpressing L6 myoblasts by using a protocol adapted from the working group of Amira Klip [21]. The negative effect on GLUT4 cycling that was observed in L6-cells was similarly reproducible in H9C2 cells, suggesting a generalisable pathology associated with GLUT4 traffic that might be constrained by reactive glucose metabolites. Based on this, H9C2KE2 is a useful model for studying the effects of chronic glucose overflow and glucotoxicity on the myocardium.

It is known that extracellular stimulation of methylglyoxal or knockdown levels of glyoxalase 1 raises the methylglyoxal concentrations, inducing the translocation of GLUT4 [6,7]. Similarly, in our model, reduced transcription of glyoxalase 1 was found under high GLUT4 presence on the surface, although higher GLO1 enzyme activity, as well as increased Nrf2 expression and phosphorylation, were detected. This “transient positive” response might be explained by the relevant strength of the detox system in reaching exhaustion, the strong supportive mechanism between the glyoxalase and the antioxidant enzymes, and the limited power of our model to stimulate the glycation process. We assume that these beneficial effects tend to disappear after chronic glucose influx, driving the glyoxalase and antioxidant systems to a breakdown, as seen in long-standing diabetes patients and at the myocardium level under huge pressure overload [22]. This conclusion is also supported by the fact that increased levels of apoptosis and necrosis, particularly in hyperglycaemic H9C2KE2 cells, were already observed in our model.

Following the metabolic changes, the TCA cycle was affected on the level of fumarate metabolism in our model. Consequently, the total protein responses were evaluated in order to understand on a more complex level how TCA cycles enzymes demand for keeping its function. However, an opposite regulation for some proteins between proteomics and Western blotting was found.

Compared to other disease models, fumarate accumulation was already associated with upregulation of the Nrf2 pathway, oxidative stress in tumour cells, cell senescence, and glucolipotoxicity [23,24,25]. Inevitably, mitochondrial activity and glucose-dependent ROS turnover were affected [23,24,25].

In the hypoxia model, the accumulation of several TCA compounds was detected by metabolomic analysis [26,27]. Whereas, particularly in the ischaemia model, the accumulation of succinate was described as a disease-specific signature, being responsible for mitochondrial ROS production during reperfusion [28]. This latter study also proved that succinate accumulation was influenced by fumarate overflow, which was probably in charge of reversing the succinate dehydrogenase reaction [28]. This fumarate overflow was suspected to arise from purine nucleotide metabolism and from the partial reversal of the malate/aspartate shuttle in this scenario. Therefore, during reperfusion, re-oxidation of succinate generates ROS in the mitochondrial complex I [28]. Together, these findings support the dual role of fumarate in inducing ROS and stimulating antioxidative pathways via Nrf2-stimulation.

Similarly, we observed increased ROS formation and ROS-associated proteins as a possible result of an inefficient TCA cycle activity. Our possible explanation is that the accumulation of fumarate concomitantly suppresses the activity of succinate dehydrogenase, generating even more ROS as a vicious cycle. As a consequence, pyruvate carboxylase (PC) fails to recompense the oxaloacetate loss in the TCA cycle, due to the increased glucose influx (in H9C2KE2 cells). Then, PC is downregulated to nearly 50% when compared with H9C2KE2 normoglycaemic cells. Besides this, pyruvate dehydrogenase complex was upregulated and, via increased phosphorylation, blocked in its activity, preventing the processing of pyruvate to acetyl-CoA. This results in pyruvate accumulation, as shown by our measurements. However, in a normal condition, WT cells can restore the pyruvate levels and refill the TCA cycle, except for H9C2KE cells, which, at this level, already lost the restoring capacity. Thus, the TCA cycle of H9C2KE cells runs out of compounds. It was not our intention to analyse the effect of TCA agonists/antagonists within this experimental setting; however, one might assume that, besides tight glucose control, TCA activation might reduce fumarate levels and thus reduce symptoms of oxidative stress.

Interestingly, this is the first study that deeply investigated levels of proteins from the TCA cycle in a cell culture model of DC onset. Therefore, our findings indicate the need for improving glycaemic control as a basis for the prevention of the disease. Particularly in the myocardium, impaired GLUT4 traffic induced by reactive glucose metabolites and glucose-induced loss of function of the TCA cycle can be observed as an essential mechanism. Before this present study, a clinical study was able to detect a high level of TCA metabolites in the plasma of patients with a high risk of heart failure; however, diabetes was not the only risk factor of these patients [29]. In addition, after knowing how the TCA cycle responds during long-term hyperglycaemia, future studies can better address the therapy based on intermediate metabolites from the TCA cycle for treating DC.

## 4. Conclusion

**Figure 7:**
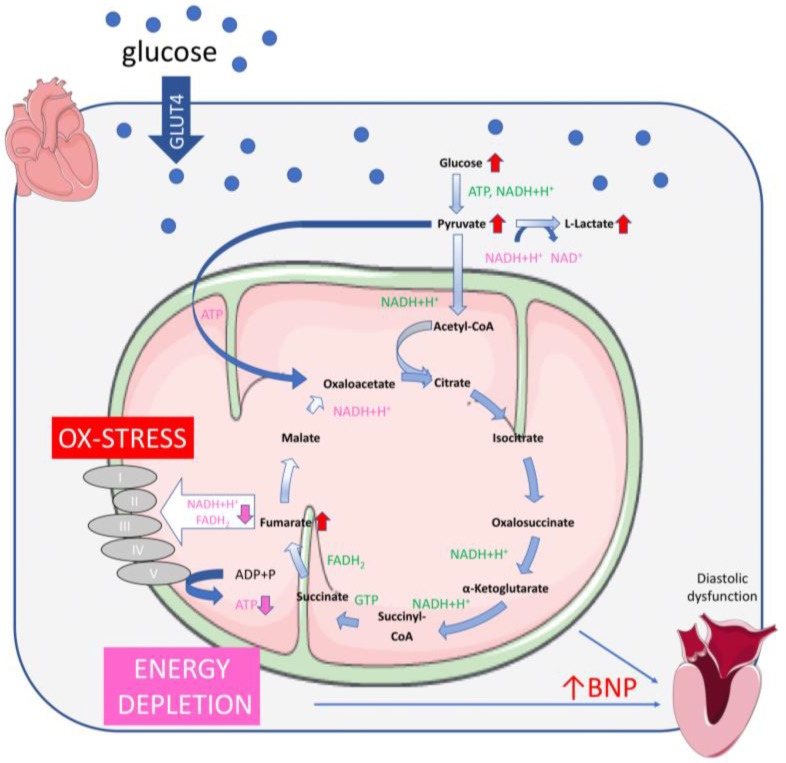
Overview on metabolic stress in H9C2KE2 cells induced by glucose overflow; delivered products in green, demanded products in red. Within the TCA cycle, reduced amounts of fumarase induce fumarate overflow and lack of malate and following compounds of the cycle such as oxaloacetate. Oxaloacetate might be refilled by the pyruvate carboxylase at the expense of ATP. With the TCA cycle running out of substrates, the respiratory chain is lacking protons used for ATP production. Accumulation of fumarate is inducing oxidative stress via blocking the succinate dehydrogenase. Anaerobic glycolysis is pronounced as detected by increased L-lactate production. As a consequence of energy deprivation from the TCA cycle, increased BNP levels are released on the heart, provoking diastolic dysfunction. Observed findings are depicted with red arrows or lines, estimated consequences are shown in pink. The figure was drawn using pictures from “Servier Medical Art” (http://smart.servier.com/; accessed on 15 April 2022).

In the classical view of DC, the myocardium suffers from a lack of energy although and due to the surplus of both glucose and fatty acids. Both compounds hamper each other’s metabolism, and thus the heart runs out of fuel [30]. In summary, our findings demask how the metabolic stress induced by hyperglycaemia can affect the TCA cycle at the fumarase level, resulting in a probable loss of NADH. Hereby, at the level of heart cells, the TCA cycle may run out of components that are necessary for functioning, resulting in inefficient energy production and a lack of fuel (Figure 7). Our model might resemble the beginning of the process of DC, induced by glucose overflow as happens in diabetes mellitus at early stages. As the observed effects are insulin-independent, our model might apply for both types of diabetes, type 1 and type 2, providing a more prospective opportunity for the development of new therapies.

## 5. Materials and Methods

### 5.1. Plasmid Constructions

The ratGLUT4-cDNA containing a fourteen amino acid epitope of human c-myc (5′-CCCGCAGAGGAGCAAAAGCTTATTTCTGAAGAGGACTTGCTTAAG-3′) within its first exofacial loop (according to [21]) was amplified using specific oligonucleotides introducing the BamHI/XhoI restriction site for cloning into pcDNA3.1(+) vector (cat. No. #V79020, Invitrogen, Carlsbad, CA, USA). After successful cloning, H9C2 cells were transfected using Lipofectamine^TM^2000 transfection reagent (cat. No. #11668-019, Invitrogen, Carlsbad, CA, USA). Positive transfectants were selected by geneticin-resistance. Cells were plated in adequate dilution for single-cell cultivation and positive transfectants were identified by gene sequencing [31].

### 5.2. Cell Culture

H9C2 cells (cat. No. #CRL-1446, ATCC, Rockville, MD, USA) were used for establishing a new cell culture model of diabetic cardiomyopathy. They were initially maintained in DMEM (cat. No. #11966-025, Gibco, Grand Island, NY, USA) supplemented with 10% (*v/v*) foetal bovine serum (FBS superior, cat. No. #S0615, Sigma-Aldrich, St. Louis, MI, USA), HEPES (25 mM, cat. No. #1563-056, Gibco, Grand Island, NY, USA), sodium bicarbonate (2%, cat. No. #25080-060, Sigma-Aldrich, St. Louis, MI, USA), non-essential amino acids (MEM-NEAA, 1%) (cat. No. #11140-035, Gibco, Grand Island, NY, USA), and glucose (Glucose 50% pro infusion, Fresenius Kabi, Bad Homburg v. d. Hoehe, Germany) to a final concentration of 20 mM glucose.

After that, cardiomyoblast-derived cells were exposed to different glucose concentrations, which were known concentrations for mimicking a normoglycaemic stage (20 mM) or a hyperglycaemic stage (30 mM). The cells were incubated at 37 °C in a humidified atmosphere of 5% CO_2_, and they were used when the confluence reached around 70%–80%. H9C2KE2 cells were maintained in geneticin G418 (1.2%, cat. No. #10131-027, Gibco, Grand Island, NY, USA)-containing medium. Additionally, all the mediums were supplemented with Penicillin-Streptomycin (1%, cat. No. #15140-122, Gibco, Grand Island, NY, USA). To keep glycaemic status, cells were frozen in 20 or 30 mM glucose-containing medium, respectively.

### 5.3. Hyperglycaemic Stage Measurements

Rat cardiomyocytes stably overexpressing GLUT4 (H9C2KE2) were exposed to normoglycaemic (20 mM glucose) and to hyperglycaemic (30 mM glucose) culture conditions for the long-term (9 months). After this time, the glucose uptake was determined by measuring the uptake of 2-(N-(7-nitrobenz-2-oxa-1,3-diazol-4-yl)amino)-2-deoxyglucose (2-NBDG) (cat. No. #N13195, Invitrogen, Carlsbad, CA, USA). For this measurement, cells were incubated with 2-NBDG in a final concentration of 100–200 µg/mL in glucose-free medium for 1 h. Then, the cells were centrifugated for 5 min at 400× *g* at room temperature. The supernatant was removed, and the pellets were washed with 200 µL of cold PBS. At this point, PI (1/1000, cat. No. #P4170, Sigma-Aldrich, St. Louis, MI, USA) was included for excluding the glucose uptake values given by necrotic cells (false positive uptake). 2-NBDG uptake by the cells was detected with fluorescent filters (excitation/emission = 488/535 nm). Propidium iodide fluoresces in necrotic cells only with Ex/Em = 488/650 nm. Measurements were taken by flow cytometry.

In parallel, glucose consumption and lactate production of each group of cells were also measured in cell culture supernatant via blood analyser (ABL800 FLEX radiometer, Krefeld, Germany).

During this time, the morphology of the cells was determined using an inverted microscopy (Eclipse TE2000-U, Nikon, Amsterdam, The Netherlands). The granularity and size were measured by flow cytometry (FC500, Beckman Coulter, Krefeld, Germany) and the total protein quantification was measured by bicinchoninic acid assay (cat. No. #B9643, Sigma-Aldrich, St. Louis, MI, USA).

### 5.4. Glyoxalase-1 Activity Assay

Glo1 activity was measured in H9C2KE2 cells according to Arai et al. [32]. Briefly, cells were washed with PBS and incubated for 3 min at 37 °C with trypsin-EDTA solution (cat. No. #9002-07-7, Sigma-Aldrich, St. Louis, MI, USA). FCS-containing medium was used to stop the trypsin reaction. Then, cells were centrifugated (3 min at 350× *g* and 4 °C), washed with 4 °C cold PBS, centrifuged, and resuspended in 50 μL of 10 mM sodium phosphate buffer (pH 7.0, including protease inhibitor, cat. No. #P2714, Sigma-Aldrich, St. Louis, MI, USA). Afterwards, repeated freeze–thaw cycles followed by ultrasonic treatment for 20 s were used for homogenising the samples. The lysate arising from these steps were centrifuged for 30 min at 4 °C at 3220× *g* and the supernatant was used for the assay. Total protein concentration was determined using BSA (cat. No. #B9643, Sigma-Aldrich, St. Louis, MI, USA) as described above. A total of 4 μg (in 5 µL buffer) protein was loaded in each well of the 96-well UV microtitre plates. Blank buffer (containing 50 mM NaH2PO4, cat. No. #1065801000, Merck, Darmstadt, Germany) and reaction buffer (containing 50 mM NaH2PO4 (pH 6.6) [cat. No. #1065801000, Merck, Darmstadt, Germany], 2 mM GSH (cat. No. #G4251, Sigma-Aldrich, St. Louis, MI, USA), and 2 mM MG (cat. No. #M0252, Sigma-Aldrich, St. Louis, MI, USA) were mixed and conserved at 37 °C for 10 min prior to being incubated with samples. A total of 245 μL of blank or test reagent was added to the protein. The increase in absorbance at 240 nm was measured over time and the activity was calculated in relation to the amount of protein used. The absorbance at 240 nm was measured over a period of 30 min at a measuring interval of 1 min and at a temperature of 37 °C. The absorbance of the blank and the reaction mixture were measured based on the blank, and the reaction mixtures were determined in triplicates. In this experiment, the photometric measurement generated hemithioacetal in situ and it was performed in a microplate reader (InfiniteTM, M1000, Tecan, Maennedorf, Switzerland). The enzyme activity of GLO1 can be calculated with the following formula:

$$\mathrm{Enzymatic}\;\mathrm{activity}\;(\mathrm{Units}/\mathrm{Total}\;\mathrm{protein}\;\mathrm{in}\;\mathrm{mg})=\left(\frac{A\left(240\;\mathrm{nm}\right)}{\min}\left(\mathrm{Test}\right)\;-\;\frac{A\left(240\;\mathrm{nm}\right)}{\min}\left(\mathrm{Test}\;-\;1\right)\right)*V*D/\left(\Delta \varepsilon \left(240\;\mathrm{nm}\right)*m*d\right)$$

A240 nm/min (Test)—Measured value minus corresponding blank value;

A240 nm/min (Test − 1)—Measured value minus corresponding blank value 1 min before A240 nm/min test;

V—Volume in mL per well;

D—Dilution factor formed from the volume of the protein sample in relation to the total volume of the assay;

∆ε240 nm—Extinction coefficient: 2.86 µmol ∗ mL^−1^ ∗ cm^−1^;

M—Total amount of protein used (mg) per well;

d—Layer thickness when measured in 96-well microtitre plate (0.7 cm).

### 5.5. Mass Spectrometry

Cell lysis was carried out as described using approx. 1–2 × 10^6^ cells (>2 µg of protein) [33]. Cell pellets were resuspended in Urea buffer (30 mM TrisBase, 2 M Thiourea, 7 M Urea). For mechanical lysis, glass beads (0.25–0.5 mm and 1.25–1.65 mm) were added. Lysates were sonicated in a sonication block (Hielscher Ultrasonics GmbH, Teltow, Germany), with an amplitude of 90 and a cycle of 0.5 5× for 50 s, with rest on ice for 90 s in between cycles [34]. The resulting lysate was centrifuged (16000× *g*, 4 °C, 10 min) and the supernatant was transferred to another reaction tube. Protein concentration was determined via Bradford assay. Digestion of proteins into peptides was carried out. In brief, 10 µg of lysate were added to the digestion buffer (50 mM ammoniumbicarbonate). Samples were reduced with 15 mM dithiothreitol for 30 min at 56 °C and alkylated with 5 mM iodacetamide for 30 min in the dark at room temperature. Digestion was carried out over night at 37 °C, using trypsin as protease (ration 1:50). Digestion was stopped by acidification. Prior to mass spectrometry, the peptide concentration was determined by amino acid analysis and 200 ng of peptides were taken for the measurements [34]. Mass spectrometry was carried out as described by Plum et al. [35]. Briefly, nanoHPLC analysis was performed on an UltiMate 3000 RSLC nano LC system (ThermoFisher Scientific, Bremen, Germany). Peptides were loaded on a capillary pre-column (ThermoFisher Scientific, Bremen, Germany, 100 μm × 2 cm, particle size 5 μm, pore size 100 Å) and subsequently onto an analytical C18 column (ThermoFisher Scientific, Bremen, Germany, 75 μm × 50 cm, particle size 2 μm, pore size 100 Å). Peptide separation was achieved with a flow rate of 400 nL/min and a linear gradient up to 40% buffer B (84% acetonitrile, 0.1% formic acid). The HPLC system was online coupled to the nano ESI source of an Orbitrap Elite mass spectrometer (ThermoFisher Scientific, Bremen, Germany). The MS1 scan range was set from 300 to 2000 *m*/*z* and a resolution of 30,000. From each full scan, the Top 20 ions were selected for collision-induced dissociation (CID) fragmentation, with a normalised collision energy of 35%. The dynamic exclusion was set for 30 s. The subsequent data analysis was carried out using MaxQuant (v.1.6.10.43) [36]. The integrated Andromeda algorithm was taken to search spectra against the Uniprot rattus norvegicus reference proteome (11_2020) (UniProt, 2021) using trypsin as protease. The false discovery rate (FDR) was set to 1% for peptides (minimum length of 7 amino acids) and proteins and was determined by searching against a reverse decoy database. A maximum of two missed cleavages were allowed in the database search. Peptide identification was performed with an allowed initial precursor mass deviation up to 7 ppm and an allowed fragment mass deviation of 20 ppm. Carbamidomethylation of cysteines was set as fixed modification and oxidation of methionine as variable modification, due to sample pre-processing. Quantification was carried out using the MaxQuant Label Free Quantification (LFQ) algorithm including unique and razor peptides for quantification. For further quantification, the calculation of iBAQ values was enabled [37]. Resulting data were subsequently statistically analysed using Perseus (v. 1.6.14.0) [36]. iBAQ values were normalised by calculating the sum of all iBAQ values for each sample separately. To improve readability, iBAQ sums were divided by 1000. Single iBAQ values were subsequently divided by the respective sum (sample-wise). Resulting normalised iBAQ values were averaged for each sample group and were used for intensity-based absolute quantification of proteins.

### 5.6. Immunoblotting Analysis

All cells were initially washed with PBS (cat. No. #10010-001, Gibco, Grand Island, NY, USA). Then, proteinase inhibitor cocktail (cat. No. #P2714, Sigma-Aldrich, St. Louis, MI, USA) and phosphatase inhibitor (50×, cat. No. #524632, Sigma-Aldrich, St. Louis, MI, USA) was diluted in RIPA buffer (1% (*v/v*) NP-40 (cat. No. #74385 Fisher Scientific, Schwerte, Germany), 0.25% (*v*/*v*) Na-Deoxycholat (cat. No. #D6750, Sigma-Aldrich, St. Louis, MI, USA), 150 mmol/L NaCl (cat. No. #106.401.000, Merck, Darmstadt, Germany), 1 mmol/L EGTA (cat. No. #E5134, Sigma-Aldrich, St. Louis, MI, USA), 1 mmol/L Na_3_VO_4_ (cat. No. #S6508 Sigma-Aldrich, St. Louis, MI, USA), 1 mmol/L NaF (cat. No. #1.064.490.250, Merck, Darmstadt, Germany), and 50 mmol/L Tris (cat. No. #90903 Carl Roth, Karlsruhe, Germany), pH 7.4). The cells were manually detached using a cell scraper. An ultrasound (Ultrassound Bandelin Sonorex RK 100 SH, Berlin, Deutschland) treatment was applied 3 times, providing the samples with a temperature shock on liquid nitrogen. Then, the samples were centrifuged at 3200× *g* for 10 min in 4 °C. Protein content was measured by bicinchoninic acid assay (cat. No. #B9643, Sigma-Aldrich, St. Louis, MI, USA) using bovine serum albumin (BSA, 422351S, VWR, Darmstadt, Germany) as a standard curve. Equal amounts of total protein (10 to 20 μg, dependent on antibody) were loaded on 4–12% Bis-Tris gel (cat. No. #NP0329BOX, Invitrogen, Carlsbad, CA, USA) for electrophoresis, and transferred onto nitrocellulose membranes (cat. No. #1620115, BioRad, Hercules, CA, USA). Precision Plus Western C Standard (Cat. No. 161-0376, BioRad, Hercules, CA, USA) or precision Plus Standard Dual colour (Cat. No. 161-0374, BioRad, Hercules, CA, USA) was used as a molecular weight marker. Membranes were blocked by 5% non-fat dried milk (M7409; Sigma-Aldrich, St. Louis, MI, USA) or 5% BSA in Tris-buffered saline (BSA, 422351S, VWR, Darmstadt, Germany, TBST, cat. No. #4855.1, Carl Roth, Karlsruhe, Germany), according to the antibody needs, for 1 h at room temperature. Then, they were washed and incubated overnight at 4 °C with anti-alpha Tubulin (1:1000, cat. No. #2125, Cell Signaling, Danvers, MA, USA); anti-Fumarate Hydratase (1:5000, cat. No. PA5-22091, ThermoFisher, Waltham, MA, USA); anti-GAPDH (1:5000, cat. No. ab8245-100; Abcam, Cambridge, UK); anti-GLO1α (1:1000, cat. No. MA1-13029; ThermoFisher, Waltham, MA, USA); anti-GLUT4 (1:2000, cat. No. PA1-1065, ThermoFisher, Waltham, MA, USA); anti-Nrf2 total (detected as a double band, 1:1000, cat. No. PA5-68817, ThermoFisher, Waltham, MA, USA); anti-*p*-Nrf2 (1:1000, cat. No. PA5-67520, ThermoFisher, Waltham, MA, USA); anti-PDH (1:1000, cat. No. #3205, Cell Signaling, Danvers, MA, USA); anti-PDH phospho (1:1000, cat. No. #31866, Cell Signaling, Danvers, MA, USA); anti-PGC1 alpha (1:1000, cat. No. ab191838, Abcam, Cambridge, UK); anti-Pyruvat Carboxylase (1:500, cat. No. ab191838, ThermoFisher, Waltham, MA, USA); anti-Succinat Dehydrogenase B (1:1000, cat. No. #459230, ThermoFisher, Waltham, MA, USA); and anti-ß Actin (1:500, cat. No. #3700, Cell Signaling, Danvers, MA, USA) antibodies. Following incubation, membranes were washed and incubated with secondary ECL-anti-rabbit-HRP linked antibody (1:4000 or 1:5000, cat. No. #NA934V, Amersham, Marlborough, MA, USA) or HRP-goat anti-mouse Ig (1:4000, cat. No. #554002; Becton Dickinson, Franklin Lanes, NJ, USA). Antibodies were diluted in 0.5% BSA/TBST (BSA, 422351S, VWR, Darmstadt, Germany, TBST, cat. No. #4855.1, Carl Roth, Karlsruhe, Germany) or dry milk powder solution, depending on antibody needs. HRP substrate Western Bright chemiluminescent Substrate Quantum (cat. No. #541015, Biozym, Oldendorf, Germany) was used and recorded by a Charge Coupled Device (CCD)-camera system (Flour Chem FC3, Cell Biosciences, Santa Clara, CA, USA).

### 5.7. Quantitative Real Time PCR Analysis (qRT-PCR)

Cells were washed with ice-cold PBS and detached using an RLT buffer (cat. No. #M3148, Qiagen, Hilden, Germany) containing β-mercaptoethanol (10 µL ßME per mL RLT buffer, cat. No. #M3148, Sigma-Aldrich, St. Louis, MI, USA) for cell lysis; they were then transferred to QIAshredder columns (cat. No. #79656, Qiagen, Hilden, Germany). An RNeasy Fibrous Tissue Kit (cat. No. #74704, Qiagen, Hilden, Germany) was used for purifying total RNA and a NanoDrop 2000 (PeqLab Biotechnologie GmbH, Erlangen, Germany) was used for quantifying RNA concentrations. After reverse transcription, purified cDNA was determined via quantitative real time PCR using StepOne Plus (Applied Biosystems, Waltham, MA, USA) with fluorescent SYBR^®^ Green qPCR SuperMix-UDG (cat. No. #4367659, Applied Biosystems, Waltham, MA, USA). Oligonucleotide sequences are available in the supplementary material (Table S1).

### 5.8. Measurement of Ros Generation by Flow Cytometry

Cells were washed twice with Hank’s buffered saline solution (HBSS, pH 7.4, cat. No. #14175-053, Gibco, Grand Island, NY, USA) and 2′,7′-dichlorodihydrofluorescein diacetate (DCF-DA) (cat. No. #D6883-50MG, Sigma-Aldrich, St. Louis, MI, USA) was added. Cells were incubated at 37 °C for 30 min in the dark. Then, cells were harvested using trypsin-EDTA (0.05%, cat. No. #25300-062, Gibco, Grand Island, NY, USA), suspended in HBSS buffer, centrifuged (5 min; 400× *g*), and suspended again in 0.5 mL HBSS buffer. Fluorescence intensity was measured by flow cytometry (FC500, Beckman Coulter, Brea, CA, USA) [38].

### 5.9. Measurement of Apoptosis and Necrosis by Flow Cytometry

Cells were double-stained with an annexin A5–FITC kit and propidium iodide (PI) (cat. No. #IM3546, Sigma-Aldrich, St. Louis, MI, USA) according to the manufacturer’s instructions. The fluorescence was immediately analysed by flow cytometry (FC500, Beckman Coulter, Brea, CA, USA). The proportion of apoptotic or necrotic cells was calculated as the percentage of the number of annexin V- or propidium iodide (PI)-positive cells [38].

### 5.10. Pyruvate Assay

Pyruvate formation was quantified using a Pyruvate Assay Kit (cat. No. #MAK071; Sigma-Aldrich, St. Louis, MI, USA). Cells were homogenised in 4 volumes of pyruvate assay buffer and the samples were centrifuged at 13,000× *g* for 10 min to remove insoluble materials. Then, the supernatant was collected and deproteinised with a 10 kDa MWCO spin filter prior to addition to the reaction. Next, the reaction solution was made by addition of 50 μL of the supernatants and 50 μL of the master reaction mix. This mixture was incubated for 30 min at room temperature in the dark. Fluorescence was measured using a microplate reader (excitation/emission ~535/587 nm; InfiniteTM, M1000, Tecan, Maennedorf, Switzerland). The relative level of pyruvate in all groups was calculated and normalised to protein concentration. The control group was assigned a value of 1, and the hyperglycaemic group was then calculated relative to the control group [39].

### 5.11. Fumarate Assay

Fumarate formation was quantified using a Fumarate Assay Kit (cat. No. #MAK060, Sigma-Aldrich, St. Louis, MI, USA). Cells were homogenised and the samples were centrifuged at 13,000× *g* for 10 min to remove insoluble materials. Then, the supernatant was collected. Next, the reaction solution was made by addition of 40 μL of the supernatants and 40 μL of the master reaction mix. This mixture was incubated for 30 min at room temperature in the dark. Absorbance at 450 nm was measured using a microplate reader (SunriseTM, Tecan, Maennedorf, Switzerland). The relative level of fumarate in all groups was calculated and normalised to protein concentration. The control group was assigned a value of 1, and the hyperglycaemic group was then calculated relative to the control group [40].

### 5.12. Brain Natriuretic Peptide (BNP) Measurement

Cells were centrifugated at 1500 rpm for 10 min at 4 °C and the supernatant was collected and quantified following the manufacturer’s protocol from the BNP-32 rat ELISA kit (cat. No. #108815, Abcam, Cambridge, UK). A microplate reader (SunriseTM, Tecan, Maennedorf, Switzerland) was used for reading the samples with a wavelength of 450 nm. Correction of optical imperfections by subtracting readings at 570 nm was performed. After the measurements, the results were obtained based on the standard curve values and the multiplication of the dilution factor.

### 5.13. Statistical Analysis

Results of the experimental studies are reported as mean ± SD. Group comparisons among glucose concentration and cell type were analysed by one-way ANOVA followed by Tukey’s multiple comparison post-test. A Shapiro-Wilk test was applied for checking the normality distribution. Direct comparisons between KE2 and WT cells in different conditions were analysed by unpaired *t*-test or Mann–Whitney test, respectively. Namely, for protein expression of GLUT4, glucose cell consumption, fumarase levels, protein expression of GAPDH, protein expression of PDH1α, protein expression of Nrf2, and protein expression of PGC1α, an unpaired *t*-test for normally distributed data was applied. For GLUT4 surface, NBDG levels, GLO1 activity, pyruvate carboxylase and pyruvate levels, SDHB, fumarate, pNrf2, ROS, apoptosis, and necrosis, a Mann–Whitney test for abnormally distributed data was applied. All of these analyses were performed using GraphPad Prism version 9.0.0 for Windows (GraphPad Software, San Diego, CA, USA). *p*-values < 0.05 were considered as statistically significant. N describes independent biological experiments.

## Data Availability

The datasets generated during and/or analysed during the current study are available from the corresponding author upon reasonable request.

## Supplementary Materials

The following are available online at https://www.mdpi.com/article/10.3390/ijms23137255/s1.

## References

[1] Rubler S., Dlugash J., Yuceoglij Y., Kumral T., Branwood A., Grishman A. (1972). New Type of Cardiomyopathy Associated with Diabetic Glomeruloscierosis. Am. Cardiol..

[2] Tan Y., Zhang Z., Zheng C., Wintergerst K.A., Keller B.B., Cai L. (2020). Mechanisms of diabetic cardiomyopathy and potential therapeutic strategies: Preclinical and clinical evidence. Nat. Rev. Cardiol..

[3] Dandamudi S., Slusser J., Mahoney D.W., Redfield M.M., Rodeheffer R.J., Chen H.H. (2014). The Prevalence of Diabetic Cardiomyopathy: A Population Based Study in Olmsted County, Minnesota. J. Card. Fail..

[4] Stratmann B., Goldstein B., Thornalley P.J., Rabbani N., Tschoepe D. (2017). Intracellular Accumulation of Methylglyoxal by Glyoxalase 1 Knock Down Alters Collagen Homoeostasis in L6 Myoblasts. Int. J. Mol. Sci..

[5] Villanueva M., Michie C., Parent S., Kanaan G.N., Rafatian G., Kanda P., Ye B., Liang W., Harper M.-E., Davis D.R. (2019). Glyoxalase 1 Prevents Chronic Hyperglycemia Induced Heart-Explant Derived Cell Dysfunction. Theranostics.

[6] Engelbrecht B., Stratmann B., Hess C., Tschoepe D., Gawlowski T. (2013). Impact of GLO1 knock down on GLUT4 trafficking and glucose uptake in L6 myoblasts. PLoS ONE.

[7] Engelbrecht B., Mattern Y., Scheibler S., Tschoepe D., Gawlowski T., Stratmann B. (2014). Methylglyoxal impairs GLUT4 trafficking and leads to increased glucose uptake in L6 myoblasts. Horm. Metab. Res..

[8] Wende A.R., Schell J.C., Ha C.M., Pepin M.E., Khalimonchuk O., Schwertz H., Pereira R.O., Brahma M.K., Tuinei J., Contreras-Ferrat A. (2020). Maintaining Myocardial Glucose Utilization in Diabetic Cardiomyopathy Accelerates Mitochondrial Dysfunction. Diabetes.

[9] Liang Q., Wang B., Pang L., Wang Y., Zheng M., Wang Q., Yan J., Xu J. (2016). Application of citrate as a tricarboxylic acid (TCA) cycle intermediate, prevents diabetic-induced heart damages in mice. Iran. J. Basic Med. Sci..

[10] Choi I., Son H., Baek J.H. (2021). Tricarboxylic Acid (TCA) Cycle Intermediates: Regulators of Immune Responses. Life.

[11] Mathew A.V., Jaiswal M., Ang L., Michailidis G., Pennathur S., Pop-Busui R. (2020). Impaired Amino Acid and TCA Metabolism and Cardiovascular Autonomic Neuropathy Progression in Type 1 Diabetes. Diabetes.

[12] Schrauwen P., Hesselink M.K.C. (2018). Reduced tricarboxylic acid cycle flux in type 2 diabetes mellitus?. Diabetologia.

[13] Gaster M. (2012). Reduced TCA Flux in Diabetic Myotubes: Determined by Single Defects?. Biochem. Res. Int..

[14] Zordoky B., El-Kadi A.O.S. (2007). H9c2 cell line is a valuable in vitro model to study the drug metabolizing enzymes in the heart. J. Pharmacol. Toxicol. Methods.

[15] Stratmann B., Mattern Y., de Carvalho T.S., Tschoepe D. (2021). Glucose suicide mechanisms induced by methylglyoxal. Diabetologia.

[16] Wang X., McLennan S.V., Allen T.J., Tsoutsman T., Semsarian C., Twigg S.M. (2009). Adverse effects of high glucose and free fatty acid on cardiomyocytes are mediated by connective tissue growth factor. Am. J. Physiol. Cell Physiol..

[17] Doust J., Lehman R., Glasziou P. (2006). The role of BNP testing in heart failure. Am. Fam. Physician.

[18] Barany T., Simon A., Szabo G., Benko R., Mezei Z., Molnar L., Becker D., Merkely B., Zima E., Horvath E.M. (2017). Oxidative Stress-Related Parthanatos of Circulating Mononuclear Leukocytes in Heart Failure. Oxid. Med. Cell. Longev..

[19] Talha S., Bouitbir J., Charles A.L., Zoll J., Goette-Di Marco P., Meziani F., Piquard F., Geny B. (2013). Pretreatment with brain natriuretic peptide reduces skeletal muscle mitochondrial dysfunction and oxidative stress after ischemia-reperfusion. J. Appl. Physiol..

[20] Peng Q., Hu W.T., Su H., Yang Q., Cheng X.S. (2013). Levels of B-type natriuretic peptide in chronic heart failure patients with and without diabetes mellitus. Exp. Ther. Med..

[21] Huang C., Somwar R., Patel N., Niu W., Torok D., Klip A. (2002). Sustained exposure of L6 myotubes to high glucose and insulin decreases insulin-stimulated GLUT4 translocation but upregulates GLUT4 activity. Diabetes.

[22] Condorelli G., Morisco C., Stassi G., Notte A., Farina F., Sgaramella G., de Rienzo A., Roncarati R., Trimarco B., Lembo G. (1999). Increased cardiomyocyte apoptosis and changes in proapoptotic and antiapoptotic genes bax and bcl-2 during left ventricular adaptations to chronic pressure overload in the rat. Circulation.

[23] van der Merwe M., van Niekerk G., Fourie C., du Plessis M., Engelbrecht A.M. (2021). The impact of mitochondria on cancer treatment resistance. Cell. Oncol..

[24] Schultheis J., Beckmann D., Mulac D., Muller L., Esselen M., Dufer M. (2019). Nrf2 Activation Protects Mouse Beta Cells from Glucolipotoxicity by Restoring Mitochondrial Function and Physiological Redox Balance. Oxid. Med. Cell. Longev..

[25] Zheng L., Cardaci S., Jerby L., MacKenzie E.D., Sciacovelli M., Johnson T.I., Gaude E., King A., Leach J.D., Edrada-Ebel R. (2015). Fumarate induces redox-dependent senescence by modifying glutathione metabolism. Nat. Commun..

[26] Xie H., Xu G., Aa J., Gu S., Gao Y. (2021). Modulation of Perturbed Cardiac Metabolism in Rats under High-Altitude Hypoxia by Combination Treatment With L-carnitine and Trimetazidine. Front. Physiol..

[27] Zhang X., Liu C., Liu C., Wang Y., Zhang W., Xing Y. (2019). Trimetazidine and lcarnitine prevent heart aging and cardiac metabolic impairment in rats via regulating cardiac metabolic substrates. Exp. Gerontol..

[28] Chouchani E.T., Pell V.R., Gaude E., Aksentijevic D., Sundier S.Y., Robb E.L., Logan A., Nadtochiy S.M., Ord E.N.J., Smith A.C. (2014). Ischaemic accumulation of succinate controls reperfusion injury through mitochondrial ROS. Nature.

[29] Bulló M., Papandreou C., García-Gavilán J., Ruiz-Canela M., Guasch-Ferré J.L.M., Toledo E., Clish C., Corella D., Estruch R., Ros E. (2021). Tricarboxylic acid cycle related-metabolites and risk of atrial fibrillation and heart failure. Metab. Clin. Exp..

[30] Bugger H., Abel E.D. (2014). Molecular mechanisms of diabetic cardiomyopathy. Diabetologia.

[31] Koumanov F., Jin B., Yang J., Holman G.D. (2005). Insulin signaling meets vesicle traffic of GLUT4 at a plasma-membrane-activated fusion step. Cell Metab..

[32] Arai M., Nihonmatsu-Kikuchi N., Itokawa M., Rabbani N., Thornalley P.J. (2014). Measurement of glyoxalase activities *Biochem*. Soc. Trans..

[33] Peischard S., Ho H.T., Piccini I., Strutz-Seebohm N., Ropke A., Liashkovich I., Gosain H., Rieger B., Klingel K., Eggers B. (2020). The first versatile human iPSC-based model of ectopic virus induction allows new insights in RNA-virus disease. Sci. Rep..

[34] Krumpochova P., Bruyneela B., Molenaarb D., Koukoua A., Wuhrera M., Niessena W.M.A., Gierab M. (2015). Amino acid analysis using chromatography–mass spectrometry: An inter platform comparison study. J. Pharm. Biomed. Anal..

[35] Plum T., Wang X., Rettel M., Krijgsveld J., Feyerabend T.B., Rodewald H.-R. (2020). Human Mast Cell Proteome Reveals Unique Lineage, Putative Functions, and Structural Basis for Cell Ablation. Immunity.

[36] Tyanova S., Temu T., Cox J. (2016). The MaxQuant computational platform for mass spectrometry-based shotgun proteomics. Nat. Protoc..

[37] Schwanhäusser B., Busse D., Li N., Dittmar G., Schuchhardt J., Wolf J., Chen W., Selbach M. (2011). Global quantification ofmammalian gene expression control. Nature.

[38] Stratmann B., Engelbrecht B., Espelage B.C., Klusmeier N., Tiemann J., Gawlowski T., Mattern Y., Eisenacher M., Meyer H.E., Rabbani N. (2016). Glyoxalase 1-knockdown in human aortic endothelial cells—Effect on the proteome and endothelial function estimates. Sci. Rep..

[39] Wareham L.K., Begg R., Jesse H.E., Van Beilen J.W.A., Ali S., Svistunenko D., McLean S., Hellingwerf K.J., Sanguinetti G., Poole R.K. (2016). Carbon Monoxide Gas Is Not Inert, but Global, in Its Consequences for Bacterial Gene Expression, Iron Acquisition, and Antibiotic Resistance. Antioxid. Redox Signal..

[40] Zhou X., Xu M., Zeng W., Chen Z., Lu G., Gong Y., Finnell R.H., Xiao H., Qiao B., Wang H. (2018). Combined effects of FH (E404D) and ACOX2 (R409H) cause metabolic defects in primary cardiac malignant tumor. Cell Death Discov..

