# Supplementary material for: Chronic Hyperglycaemia Inhibits Tricarboxylic Acid Cycle in Rat Cardiomyoblasts Overexpressing Glucose Transporter Type 4
Source: Int J Mol Sci. 2022 Jun 29;23(13):7255. doi: 10.3390/ijms23137255 (PMC9266806; doi:10.3390/ijms23137255)
Supplement: Supplementary file 1 [file ijms-23-07255-s001.zip › Supplementary material_IJMS 29062022.pdf]

Chronic hyperglycaemia inhibits tricarboxylic acid cycle in rat cardiomyoblasts overexpressing glucose transporter type 4

Bernd Stratmann[1*]; Britta Eggers[2,3]; Yvonne Mattern[1]; Tayana Silva de Carvalho[1]; Katrin Marcus[2,3]; Diethelm Tschoepe[1,4]

[1] Herz- and Diabeteszentrum NRW, Diabeteszentrum, Ruhr-Universität Bochum, 32545 Bad Oeynhausen, Germany

[2] Medizinisches Proteom-Center, Medical Faculty, Ruhr-University Bochum, 44801 Bochum, Germany Centre for Translational and Behavioural Neurosciences

[3] Medical Proteome Analysis, Centre for Protein diagnostics (PRODI), Ruhr-University Bochum,44801 Bochum, Germany.

[4] Stiftung DHD (Der herzkranke Diabetiker) Stiftung in der Deutschen Diabetes-Stiftung, Bad Oeynhausen, Germany

Supplemental Figures:	3
Supplemental Table	1

Supplemental figures S1 to S3 and Table S1

Figure S1. Reactive glucose metabolite defence and cell structure changes on H9C2KE2 cells. A: L-Lactate (cell culture supernatant) (N=3); B: *GLO1* gene expression (N=3); C: Glo1 protein expression (N=3); D: D-Lactate (cell culture supernatant) (N=3); E: Cell size (N=6); F: Granularity quantification (N=6); G: Total protein quantity (mg) (N=6). All the samples were measured at least 3 times (technical repetition). Data are shown as mean±SD values described as WT20L vs. KE220L and WT30L vs. KE230L with *p<0.05, **p<0.01, ***p<0.001, or ****p<0.0001 for showing the significance. (WTL – Wild-type cells exposed for long-term/ KE2L – GLUT4 overexpressing cells exposed for long-term).

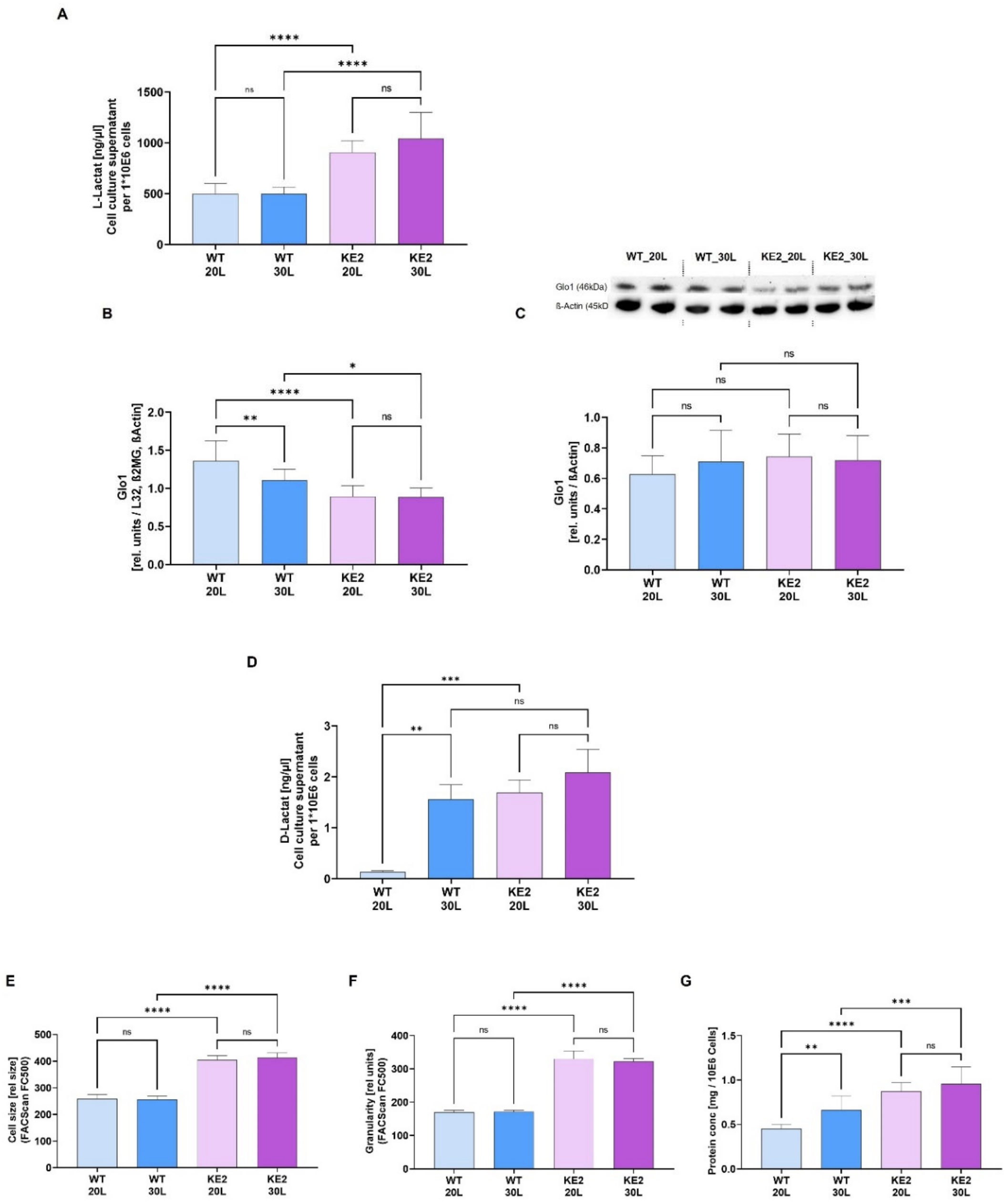

Figure S2. Glucose metabolism alteration at gene expression level. A: *GAPDH* gene expression (N=3); B: *PDH1 alpha* gene expression (N=3). Data are shown as mean±SD values described as KE220L vs. KE230L, WT20L vs. KE220L, and WT30L vs. KE230L with *p<0.05, or **p<0.01, for showing the significance. (WTL – Wild-type cells exposed for long-term/ KE2L – GLUT4 overexpressing cells exposed for long-term).

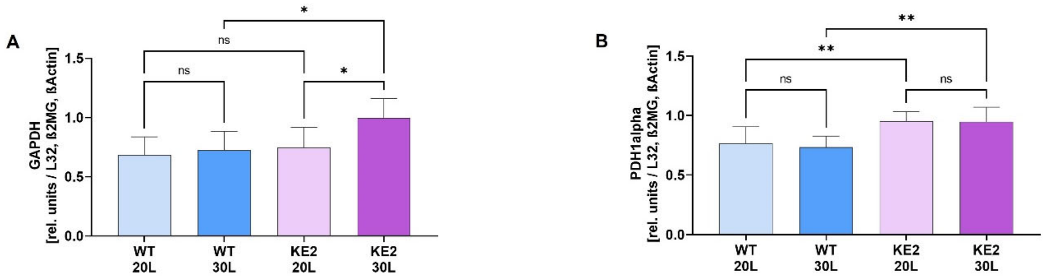

Figure S3. ROS and antioxidant response at gene expression level. A: *NRF2* gene expression (N=4); B: *KEAP1* gene expression (N=4); C: *NQO1* gene expression (N=3), D: *HO1* gene expression (N=3). Data are shown as mean±SD values described as KE220L vs. KE230L, WT20L vs. KE220L, and WT30L vs. KE230L with *p<0.05, **p<0.01, or ****p<0.0001, for showing the significance. WTL – Wild-type cells exposed for long-term/ KE2L – GLUT4 overexpressing cells exposed for long-term).

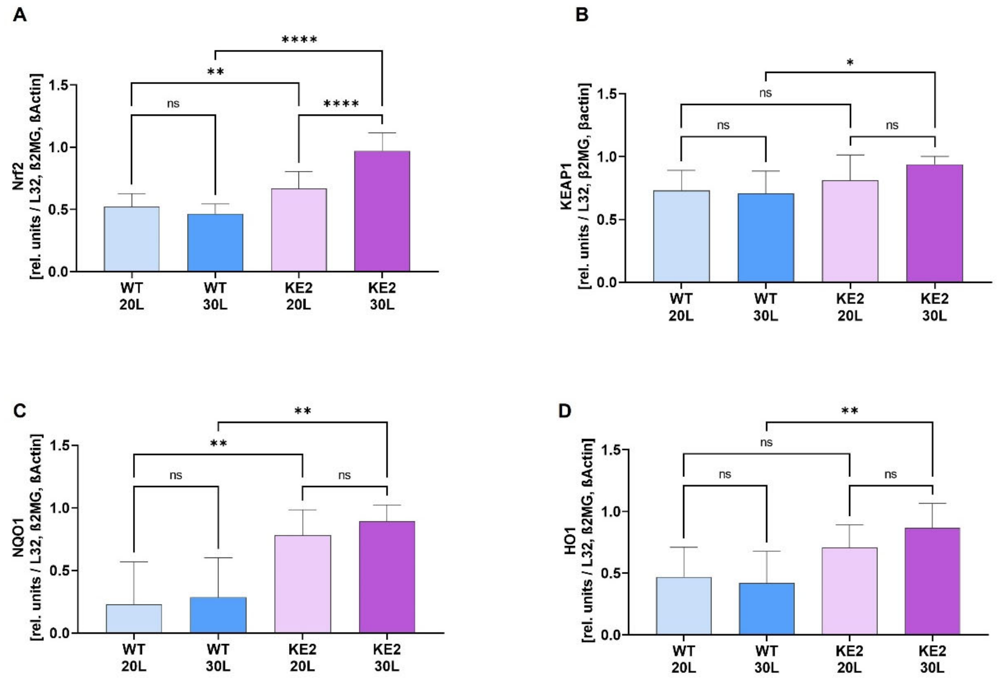

Supplemental figures S1 to S3 and Table S1

Table S1. List of PCR oligonucleotides

Oligonucleotide	DNA strand	Sequence (5'->3')
GLO1	Forward	GAT-GTC-TAC-GAG-GCC-TGT-AAG-AG
	Reverse	CCA-GTA-GCC-ATC-AGG-ATC-TTG-A
GAPDH	Forward	GTC-GTG-GAT-CTG-ACG-TGC-C
	Reverse	TGC-CTG-CTT-CAC-CAC-CTT-CT
GLUT4 cloning	Forward	AAA-AAA-GGA-TCC-GCA-ATG-CCG-TCG-GGT-TTC-CAG
	Reverse	AAA-AAA-CTC-GAG-TCA-GTC-ATT-CTC-ATC-TGG-CCC-TAA-G
GLUT 4	Forward	ACG-GAC-ACC-TTC-TCT-CTT-AGA-GCA
	Reverse	AGG-GCT-AAA-GTG-CTG-CGA-GGA-A
HMOX1	Forward	GTC AGG TGT CCA GGG AAG G
	Reverse	GGA AGT AGA GTG GGG CAT AGA
KEAP1	Forward	AGC-AGC-GTG-GAG-AGA-TAT-GAG
	Reverse	TGC-ATA-CAG-CAA-GCG-GTT-GAG
L32	Forward	GGA-GCT-GGA-AGT-GCT-GCT-GA
	Reverse	TTG-GTG-ACT-CCT-GAT-GGC-CAG
NRF2 (Nfe2le)	Forward	GCC-TGG-GTT-CAG-TGA-CTC-GGA
	Reverse	CTG-TGC-CCT-TGA-GCT-GGC-GA
NQO1	Forward	GAC ATC ACA GGG GAG CCG A
	Reverse	AGC TAC AAT ATC CGG GCT CAG
PDH1	Forward	CCT-GTC-CGA-GCA-ATT-CTT-GCA-G
	Reverse	ACG-ATA-CCG-TTG-CCG-CCA-TAG
ß2MG	Forward	CGA-GAC-CGA-TGT-ATA-TGC-TTG-C
	Reverse	GTC-CAG-ATG-ATT-CAG-AGC-TCC-A
ß-ACT	Forward	CAT-CGT-ACT-CCT-GCT-TGC-TG
	Reverse	CCT-CTA-TGC-CAA-CAC-AGT-GC
